# Community health workers at the dawn of a new era: 1. Introduction: tensions confronting large-scale CHW programmes

**DOI:** 10.1186/s12961-021-00752-8

**Published:** 2021-10-12

**Authors:** Stephen Hodgins, Maryse Kok, David Musoke, Simon Lewin, Lauren Crigler, Karen LeBan, Henry B. Perry

**Affiliations:** 1 School of Public Health, University of Alberta, Edmonton, Alberta Canada; 2 Department of Global Health, KIT Royal Tropical Institute, Amsterdam, The Netherlands; 3 Department of Disease Control and Environmental Health, School of Public Health, Makerere University College of Health Sciences, Kampala, Uganda; 4 Norwegian Institute of Public Health, Oslo Town, Norway; 5 Health Systems Research Unit, South African Medical Research Council, Cape Town, South Africa; 6 Crigler Consulting, LLC, Hillsborough, NC United States of America; 7 Independent Consultant, Washington, DC United States of America; 8 Health Systems Program, Department of International Health, Johns Hopkins Bloomberg School of Public Health, Baltimore, MD United States of America

**Keywords:** Community health workers, Community health worker programmes, Primary healthcare, Community health, Health systems

## Abstract

**Background:**

Community health worker (CHW) programmes are again receiving more attention in global health, as reflected in important recent WHO guidance. However, there is a risk that current CHW programme efforts may result in disappointing performance if those promoting and delivering them fail to learn from past efforts. This is the first of a series of 11 articles for a supplement entitled “Community Health Workers at the Dawn of a New Era”.

**Methods:**

Drawing on lessons from case studies of large well-established CHW programmes, published literature, and the authors’ experience, the paper highlights major issues that need to be acknowledged to design and deliver effective CHW programmes at large scale. The paper also serves as an introduction to a set of articles addressing these issues in detail.

**Results:**

The article highlights the diversity and complexity of CHW programmes, and offers insights to programme planners, policymakers, donors, and others to inform development of more effective programmes. The article proposes that be understood as actors within community health system(s) and examines five tensions confronting large-scale CHW programmes; the first two tensions concern the role of the CHW, and the remaining three, broader strategic issues:

1. What kind of an actor is the CHW? A lackey or a liberator? Provider of clinical services or health promoter?
2. Lay versus professional?
3. Government programme at scale or nongovernmental organization-led demonstration project?
4. Standardized versus tailored to context?
5. Vertical versus horizontal?

**Conclusion:**

CHWs can play a vital role in primary healthcare, but multiple conditions need to be met for them to reach their full potential.

Key message box 1: summary **Key findings**

- Across different settings, community health workers (CHWs) play diverse roles. Generally in large-scale programmes, CHWs have functions related to both health education and helping extend or bridge to primary healthcare services.
- CHWs fall along a spectrum from lay/volunteer to more professionalized.
- Nongovernmental organizations have played an important role in the development of CHW programmes and continue to engage with government in many public-sector CHW programmes.
- While evidence-informed models, interventions, and tools are important, for programmes to be effective the approaches used also need to be responsive to the local context.
- Over the past several decades, CHWs have played important roles in vertically delivered disease control programmes. Increasingly, CHWs are taking on broader roles in more integrated primary healthcare services.

**Key implications**

- The CHW is one actor in a complex, dynamic, primary healthcare system comprised of diverse interacting actors, each having agency, interests, perspectives, and values. Robust delivery of services at the most peripheral level of the primary healthcare system—reaching all who could benefit—requires functional systems enabling CHWs to play a constructive part and that, in turn, depends on their role being well understood and appropriately supported.

## Background

**Table:** 

Key message box 2	
The objective of this paper is to:Introduce a series of papers that seeks to draw lessons for large-scale CHW programmesHighlight the diversity and complexity of CHW programmes, and offer helpful insights to programme planners, policymakers, donors and others—grounded in experience from large-scale CHW programmes—to inform development of effective programmes, more fully realizing the potential of CHWs as a key element of peripheral-level primary healthcare systems	

The global health community is guided by the goals of achieving Universal Health Coverage (UHC) and ending preventable child and maternal deaths by 2030 [1]. Achieving these goals will require strengthened primary healthcare (PHC), which—in turn—will require well-supported CHWs . WHO and the World Health Assembly in 2016 called for renewed efforts to realize the potential contribution of CHWs as members of multi-professional PHC teams [2]. In 2018, WHO released a new guideline—based on systematic reviews of existing literature and programme experience—for health policy and systems support to optimize CHW programmes [3, 4]. This guideline offers specific guidance on: selection, pre-service education, certification, supervision, remuneration and career advancement, planning, community embeddedness, and health systems support. In 2019, the World Health Assembly urged Member States to integrate CHWs within their health systems and provide them the necessary support to deliver safe and high-quality care, drawing on insights from the new WHO guideline [5].

The COVID-19 pandemic has drawn attention to how important it is to have a robust health workforce at the community level, available both to ensure continued delivery of key PHC services and to play a role specific to the pandemic response, including—depending on the setting—education, prevention, screening, case detection, contact tracing, promoting immunizations, and faciltating linkages with higher levels in the system [6]. For decades now, the relevance, effectiveness, and acceptability of CHW programmes have been debated, and ensuring the funding needed for these programmes to achieve good performance has generally not been a priority for governments or for donors. Over the past decade, only 2.5% of total official development assistance for health has been directed to CHW programmes and, of that, two-thirds has been earmarked for specific disease control programmes [7].

In low- and middle-income countries (LMICs), CHW programmes have often been no more than a half-hearted response from governments to what they viewed as a time-limited problem—the unmet health needs of hard-to-reach communities, including in rural and peri-urban areas. In the minds of many policy-makers and experts, CHWs will no longer be needed once more sophisticated health services are available.

In 1988, Gill Walt concluded that “unless adjustments are made, CHW programmes will drift towards demise, not because CHWs themselves cannot deliver, but because the support that makes them effective is, in general, absent” [8]. Much the same could have been said in 1998, in 2008, or in 2018. However, the notion of CHW programmes as a cheap solution to a temporary problem in low-resource settings is increasingly recognized as a fallacy, for several reasons:

1. Functional CHW programmes are not necessarily cheap. To operate effectively, at scale, substantial numbers of CHWs are required; and high-performing programmes require continuous, robust supervisory and logistical support.
2. CHWs can do far more than close temporary gaps. In addition to roles they have traditionally played in maternal/child health services, CHWs have made important contributions in epidemic response, for Ebola in West Africa [9] and, now, for COVID-19 [10]. Increasingly, CHWs are engaged in the detection and management of HIV [11], tuberculosis (TB) [12], chronic diseases (especially hypertension, diabetes and mental illness) [13], palliative care, and medical and social support for the elderly. There is every reason to believe that in the coming decades the CHW’s role will evolve, and they will also be essential in helping health systems reach their full potential in improving population health.
3. CHW programmes are also relevant to higher-resource settings. CHWs in high-income countries are making important contributions to address health needs [14, 15]. It could be argued that CHW programmes will need to be an essential element—if not the foundation—for health systems in all countries regardless of their socioeconomic status and level of health system sophistication, if these systems are to fully meet the health needs of the populations they serve.

In 2014, several of the authors of this article contributed to the development of an online book, developed in response to a resurgence of interest in CHWs at that time, entitled *Developing and Strengthening Community Health Worker Programs at Scale: A Reference Guide and Case Studies for Program Managers and Policymakers* [16]. They were concerned that the cycle seen over the period from the 1970s to the 1990s might recur. Over that period there was at first renewed interest in CHWs, which was followed by inadequately financed and poorly planned programmes, causing weak programme performance and the eventual abandonment of the programmes altogether, as happened with the Village Health Guides programme in India [17] and other programmes.

Table 1 (below) lists the major national CHW programmes in LMICs included in a newly published compendium of case studies [18]. These programmes have over 8 million CHWs, although this is certainly not an exhaustive listing of CHW programmes; even in these countries, there are categories of health workers that could be considered CHWs that are not included. With the exception of most CHWs in South Africa and those working for Building Resources Across Communities (BRAC) in Bangladesh, who are employed by nongovernmental organizations (NGOs), all of the CHW cadres described in the compendium work within ministry of health (MOH) programmes. In this series, we will draw lessons particularly from the programme experience documented in these case studies.

**Table. 1:** Major CHW programmes in LMICs [18]

Country	Number of CHWs	Name of CHW cadre
Afghanistan	27,000	Community health workers
Bangladesh	20,000	Government family welfare assistants
	15,000	Government health assistants
	47,000	BRAC *shasthya shebikas* and *shasthya kormis*
Brazil	265,000	Community health agents
Ethiopia	40,000	Health extension workers
	3,000,000	Women’s Development Army volunteers
Ghana	3,000	Community health officers
	19,000	Community health volunteers
India	219,000	Auxiliary nurse midwives/multipurpose health workers
	971,000	Accredited social health activists
	1,300,000	*Anganwadi* workers
Indonesia	500,000	*Kaders*
Iran	61,000	*Behvarzs* and their urban equivalents, *moraghebe-salamats*
Kenya	2,000	Community health extension workers
	86,000	Community health volunteers
Liberia	3,800	Community health assistants and community health service supervisors
Madagascar	20,000	*Agents communautaires*
	20,000	*Agents communautaires de nutrition*
Malawi	17,000	Health surveillance assistants and senior health surveillance assistants
Mozambique	5,000	*Agents polivalentes elementares*
Myanmar	24,000	Auxiliary midwives
	15,000	Community health workers
	15,000	Malaria volunteers
	6,000	TB volunteers
Nepal	52,000	Female community health volunteers
Niger	2,000	*Agents de santé communautaire*
	5,000	*Relais* (volunteers)
Nigeria	43,000	Community health extension workers
	75,000	Volunteer village health workers
Pakistan	100,000	Lady health workers
Rwanda	45,000	*Binômes* and *animatrices de santé maternelle*
Sierra Leone	14,000	Community health workers and peer supervisors
South Africa	33,000	Community health workers
	70,000	NGO-supported CHWs (mostly for HIV/AIDS work)
Tanzania	12,000	Community health workers
	41,000	Volunteer community health workers
Thailand	1,000,000	Village health volunteers
Uganda	179,000	Village health team members (volunteers)
Zambia	2,000	Community health assistants
Zimbabwe	10,000	Village health workers

Now, in 2021, we have written this series, titled “Community Health Workers at the Dawn of a New Era”, to inform the development and strengthening of large-scale CHW programmes, and to encourage the growth of new programmes in the future. This set of papers is similar to the systematic reviews commissioned in support of development of the 2018 WHO guidelines in that it is intended to provide guidance for the development of national CHW programmes, but it differs in three important respects:

1. It draws not only on peer-reviewed journal articles but also on grey literature sources, including the recently released 29 CHW case studies [18], as well as other recent publications (from both the peer-reviewed and the grey literature) released since the 2018 WHO guideline was written. This series draws on insights from the field from public health professionals with long experience with large-scale CHW programmes.
2. Its scope extends considerably beyond policy and systems supports and includes detailed exploration of broad substantive issues not covered in the 2018 WHO guideline, as shown below.
3. It explicitly addresses the need to accelerate progress in achieving the 2030 global health goals, especially for those goals for which the contribution of CHWs is critical.

The authors believe the tide has turned; we are seeing countries engaging in these programmes with increased political support, greater commitments of financial resources, and a more robust integration of CHWs within the PHC system. This turnabout holds the promise of more effective programmes and services, and ultimately greater impact on population health outcomes.

The papers in the series include the following:

1. Introduction: tensions confronting large-scale CHW programmes (this paper)
2. Planning, coordination, and partnerships [19]
3. Governance [20]
4. Financing [21]
5. Roles and tasks [22]
6. Training [23]
7. Supervision [24]
8. Incentives and remuneration [25]
9. Relationships with health systems and communities [26]
10. Performance and performance assessment [27]
11. Conclusions and leading the way to Health for All [28]

**Fig. 1:**
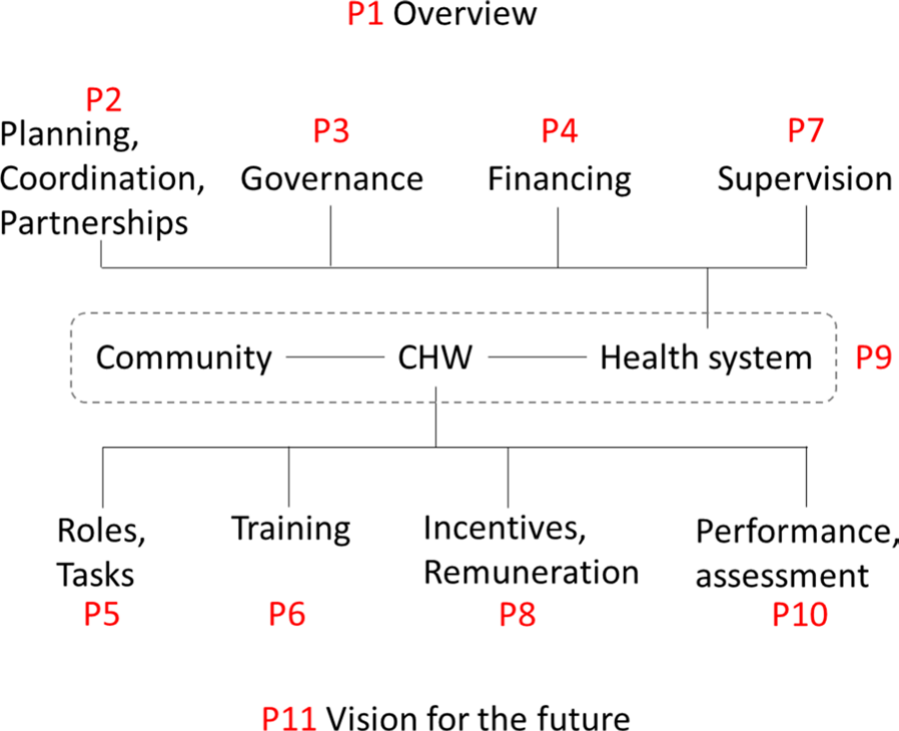
Conceptual framework for the articles included in the supplement on “Community Health Workers at the Dawn of a New Era”, grouped in relation to health systems considerations and factors relating more directly to the CHW. “P” refers to the paper in the supplement, and the number following it is the number of the paper in the supplement. The references for all the papers are in the body of the paper

The set of papers is organized conceptually in Fig. 1.

These papers build on two previously published papers in *Human Resources for Health* focused on issues facing large-scale, generally national, CHW programmes [17, 29]. This paper, the first in the set, is a commentary, drawing not only on the peer-reviewed literature but on the case studies referenced above, other grey literature, and the authors’ experience with large-scale CHW programmes.

### The community health system and its actors

In publications about CHWs, often the context and the systems of which CHWs are a part—notably, the peripheral PHC services—are relatively neglected, giving a decontextualized picture of the role and function of CHWs. A blinkered perspective on the part of those working with these programmes will result in less appropriate policy, plans, and programme designs and, therefore, in services that less adequately meet the needs of the populations they are intended to serve.

Schneider et al. [30] explain that “CHW programs interface with both the formal health system (requiring integration) and community systems (requiring embedding) in context-specific and complex ways… [D]ebates on community-based delivery of services to achieve Universal Health Coverage could more properly reflect the emerging systems perspective, by widening the focus from a cadre to the community health system as a whole.” They describe “community health systems” as including formal and informal, community-based and government actors and service providers, who work in specific contexts shaped by local histories, economic and political systems, and sociocultural norms. Kok et al. [31] also point to the intermediary position occupied by CHWs, belonging to both the community and the formal healthcare system. This and earlier published work [32] stress the often neglected, so-called soft characteristics of community health systems: ideas, interests, relationships, power, values, and norms, and how these soft characteristics influence perceptions of support, respect, competence, honesty, fairness, and recognition. We explore these issues further in Paper 9 of this series, which focuses on the relationships of CHWs with the health system and the community [26].

**Fig. 2:**
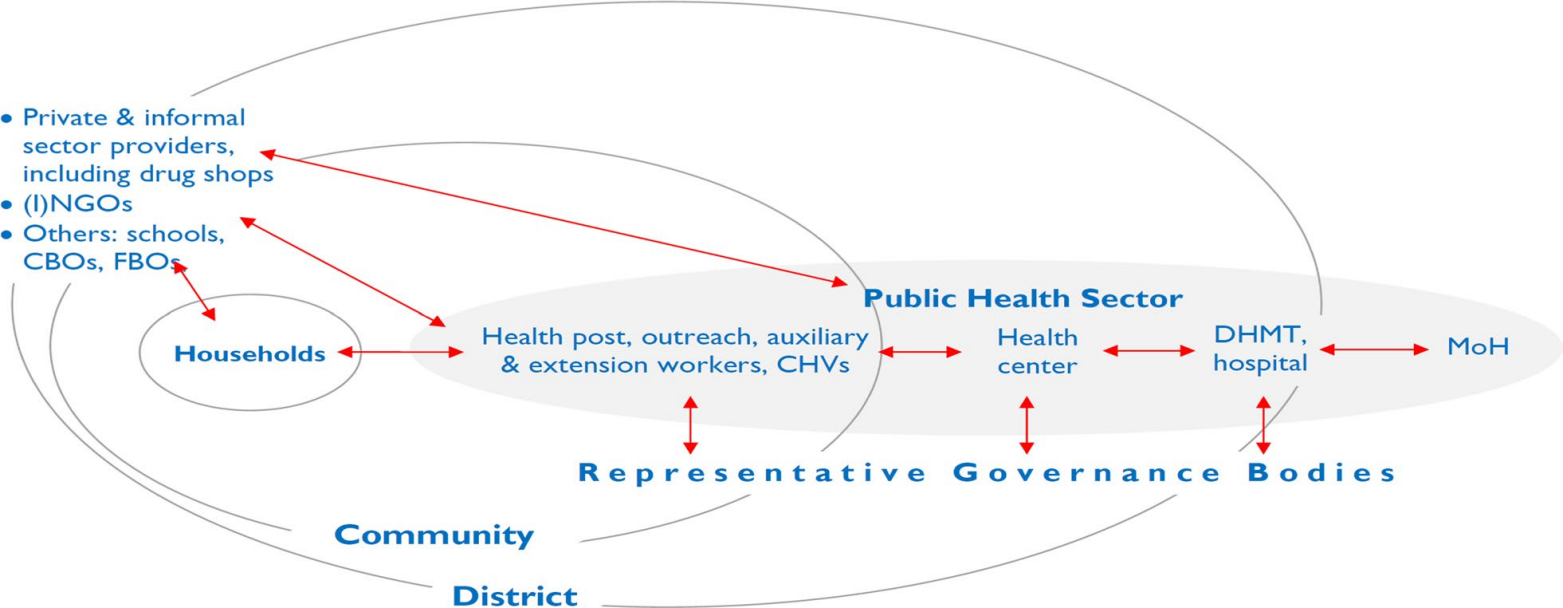
Elements and relationships in the work of the peripheral health system (public, private, and household). *(I)NGOs* international and national NGOs, *CBOs* community-based organizations, *FBOs* faith-based organizations, *CHVs* community health volunteers, *DHMT* district health management team, *MOH* Ministry of Health. Relationships indicated in red

To better understand CHWs, it is helpful to begin by considering the CHW *within the context of the community (s)he serves* as well as *within the programmatic and service delivery context in which (s)he functions*. CHWs work with a wide range of other cadres in their setting. The needs and requirements of each cadre, each manager, each actor in the peripheral PHC system, must be adequately addressed in order for this “ecosystem” to function optimally. Interaction among these system elements influences the performance of particular actors, including CHWs, and the evolution of these dynamics over time. Figure 2 is offered as a simplified schema, illustrating key actors at the most peripheral level of the PHC system along with the relationships and interactions among them.

As shown in Fig. 2, the *household* has a primary role in the production of health [33], in part through its interaction with the peripheral health system. The *community* (consisting of complex systems of its own) overlaps here with the peripheral level of the government healthcare system. The community has a fundamentally important role to play in establishing conditions within the community that may be more or less favourable to attaining better health; furthermore, community members can also play an important role in supporting and holding accountable those providing health services, including CHWs.

The most *peripheral tier of the PHC system* often includes fixed structures below the level of health centre. They go by various names, including dispensary, subcentre, community clinic, and health post. In many countries, some services are offered through periodic outreach. Staffing can include CHWs and volunteers with varying profiles. In this paper, we will consistently use a typology consisting of three broad categories, which we will explain later in the paper. They include what we will refer to as:

- auxiliary health workers,
- health extension workers (HEWs), and
- community health volunteers (CHVs).

In many CHW programmes, auxiliaries or HEWs are responsible for supervising CHVs [24, 34]. If the CHV has tasks involving community-based distribution or case management of childhood illness, supervisory contacts can be the principal means of resupplying CHVs with programme commodities. Issues of supervision are explored further in Paper 7 of this series [24]. These contacts also provide an opportunity to systematically gather data on service delivery by CHVs. HEWs themselves may be supervised by a health worker based at the health post or a higher-level facility. In some programmes, there are dedicated supervisors who spend all or most of their time supervising HEWs (or CHVs). In other settings, the supervisory role is assumed by staff who have other management and clinical functions [24, 34]. In many countries, there are entities at the district level (or equivalent), such as district health management teams, with technical and managerial oversight responsibility for peripheral-level PHC services, including the work of CHWs (including supervision, monitoring/reporting, logistics, and finance).

Settings with multiple tiers of CHWs (including volunteers) can be understood as dual- or multi-tier systems [35], as explored further in Paper 5 in this series [22]. Examples include: Ethiopia’s HEWs and Women’s Development Army volunteers, Niger’s *agents communautaires* and *relais* volunteers, and Nepal’s auxiliary health workers and female community health volunteers (FCHVs).

Staff in the peripheral tier of the PHC system may have oversight from health facility committees or boards [9]. In some instances, local bodies have authority over financial and hiring/firing decision-making. In Fig. 1, *representative governance bodies* include entities such as village health committees, development committees, health facility community boards, and local and district government to which local health service providers are accountable. As Schaff et al. point out [36], accountability can be understood in vertical terms—up through a hierarchical management structure and down to the population to be served (accountable to locally representative governance bodies of the types noted).

In many settings, private providers play an important role, particularly in sickness care. This may include both formally recognized health professionals and uncredentialed informal providers, such as traditional healers, traditional birth attendants, and drug sellers [35–37]. In some country settings, government and development partners have engaged the medicine shop sector using social marketing and social franchising models that press medicine shop proprietors and staff into service to pursue public health goals, such as improving the availability (and sometimes quality) of family planning and sick-child care services by dispensing contraceptive products, and oral rehydration salts and zinc for diarrhoea. In some instances, although not officially recognized, government PHC workers supplement their incomes in private practice. This includes extension and auxiliary health workers operating medicine shops or working privately as “village doctors” [38].

Perhaps the most paradigmatic role of the CHW is interacting with members of the community in either the CHW’s or the beneficiary’s home, as we explore further in Paper 9 in this series on relationships of CHWs with communities [26]. Two prominent types of service offered during such “doorstep” or “domiciliary” contacts are counselling/health education and dispensing health-related commodities. Their role may also entail assessing aspects of their beneficiary’s health, well-being, or household health-related practices (for example, breastfeeding or correct use of an insecticide-treated bed net). CHWs may have direct contacts with *all* households in their catchment area, or only with households *targeted* because of certain demographic characteristics, for example, those households with a pregnant woman, a child younger than 2 years of age, or someone with a specific disease (TB or HIV). CHWs are commonly an important feature of TB and HIV programmes, providing adherence support, often through home visits.

CHW outreach services may be provided in various locations in the community, such as schools or other public places such as markets. This is a commonly used strategy for routine immunization, family planning, and antenatal care. In many programmes, such outreach services are offered on a monthly basis in different locations in a health facility’s catchment area. In many countries, CHWs are also involved in periodic, campaign-style outreach activities, offered on an annual or semiannual basis, during which immunizations or key health commodities are administered or dispensed. We discuss these modalities of service delivery further in the section below on vertical versus horizontal organization of services.

In some programmes, CHWs have responsibility for a specified number of households (and the associated geographic area covered). The number of households they are expected to cover can be an important determinant of programme performance [39]; programmes in which local CHWs are spread too thinly will be unable to achieve good population coverage for prioritized interventions for which they are responsible.

To the degree that CHWs are involved in delivery of services that depend on commodities, robust logistics systems (another element of the community health system) are an absolute requirement for both programme effectiveness and credibility with the intended users of community health services. In all but 9 of the 29 case studies of national CHW programmes included in the recently published compendium mentioned earlier [18], inconsistent supply of programme commodities was noted as a significant threat to performance. When commodities are not available on a regular basis, communities lose confidence in CHWs and, in turn, CHWs are less satisfied with their work.

In summary, a CHW is one actor in a complex dynamic PHC system comprised of diverse actors with agency, interests, perceptions, and values, in interaction with each other. Robust delivery of services at the most peripheral level of the PHC system—reaching all who could benefit—requires functional systems enabling CHWs to play a constructive part, and that, in turn, depends on their role being well understood and appropriately supported.

## Tensions confronting CHW programmes

Rich or poor, essentially all societies fall short with regard to eliminating health and social disparities and ensuring robust, well-coordinated, comprehensive healthcare for all. It is widely recognized that having an actor at the interface between the community and the healthcare system can be part of the solution to this problem, and hence, the perennial interest in CHW programmes.

Recently, important new recommendations on policy and systems support for CHWs have been released [3, 4], drawing on published evidence [2, 3, 34]. Indeed, organizations such as WHO seek to provide guidance based on the best available evidence on effectiveness and, increasingly, on considerations such as acceptability and feasibility. Relative to their importance for health systems, the body of research on large-scale CHW programmes has been modest (although there have certainly been many published small proof-of-concept trials). A range of research and evaluation methods is required to generate the evidence needed to develop large-scale effective community health services, including robust, pragmatic implementation research on large-scale programmes. As stated in the 2018 WHO guideline:

In calling for additional research on the topic, it is important to recognize that, while more methodologically robust evidence is needed, it is probably unrealistic to envisage that there would be large-scale RCTs [randomized controlled trials] to address, from a pure effectiveness perspective, all the persisting evidence gaps [particularly with regard to systems supports required for effective programmes]. Furthermore, RCT design is relatively unhelpful in providing insights into the dynamics of complex programmes. More useful would be comprehensive, critical programme case studies. [3], p. 66

Drawing lessons from such programme case studies, this article and the series of which it is a part seek to direct attention to key considerations for building strong community-based PHC services. These papers focus on programmes in LMICs, primarily serving rural areas,^1^ using cadres ranging from part-time volunteers to paid, full-time, para-professional health workers.

This paper considers several important questions or issues facing those interested in delivery of services at the most peripheral level of the PHC system, which we are characterizing as “tensions”. The authors have framed these issues as tensions in the hope that this will aid policy-makers and programme developers in avoiding overly simplistic solutions and will, instead, give due attention to sometimes under-recognized tradeoffs that need to be made in optimizing for strong, effective programmes. The first two sections of the paper focus more specifically on the CHW, and the following three, on broader issues with PHC and CHW programmes.

## 1) What kind of a role should the CHW play?

**Table:** 

Key message box 3	
Although in most public-sector CHW programmes it is not expected that CHWs play a role of sociopolitical change agent, it is common that they act as *intermediaries* between health services and the community and play a role in health education and health promotion	

### A lackey or a liberator? Provider of clinical services or health promoter?

Some 40 years ago, David Werner posed the provocative question: is the CHW a “lackey” or a “liberator”? He was highlighting the distinction between extending health services and being a catalyst for social change [40, 41]. It is clear from his choice of words that Werner had ideological reasons for preferring one over the other. Colvin has drawn attention to this same tension using the less politically charged language of “extension agent” versus “agent of change” [42]. Even now, this tension has not been altogether resolved. Dating back into the nineteenth century, there has been divergence between those who see public health as a technical and biomedical enterprise focused on disease control and those who see it as a sociopolitical effort with a broader focus on well-being and on its social and economic determinants (for example, see the work of Rudolf Virchow [38]). So it is not surprising that this tension is also evident in relation to the roles CHWs should be playing. Should CHWs be public-sector employees or activists within a people’s movement? Should they be focused on provision of biomedical interventions or on advocating for the vulnerable within their communities?

In practice, most national public-sector programmes have focused on the narrower service delivery role of CHWs. Nevertheless, in many programmes, there remains an expectation that CHWs act as intermediaries between communities and health services, and that at least part of their work consists of health education or health promotion, encouraging behavioural (if not sociopolitical) change. This role as change agent (as health promoter) is often undervalued by both governments and communities, compared to biomedical services (e.g., sick-child care, family planning, immunization, dispensing mosquito nets). Paper 5 in this series, on CHW roles and tasks, explores these issues further [22].

Policy-makers and planners have responded to the shortage of trained health professionals in underserved areas by shifting clinical tasks that would normally be done by physicians or nurses [41] to health auxiliaries, extension workers, and other types of CHWs [34]. This may include assessment and treatment decisions for illness care as well as certain procedures, such as administering injections (typically vaccines and injectable contraception). Task-shifting may be a sound choice in settings where there is a shortage of health professionals with more advanced training. Indeed, being assigned such functions is often motivating to CHWs. However, in many instances, physician and nursing associations have opposed shifting tasks to CHWs, citing concerns about safety and lack of necessary skills, but, arguably, being more concerned about protecting a monopoly provider role, even when very few of their members are interested in serving in rural areas [9, 32]. In settings where there has been increasing professionalization and increasing numbers of health worker cadres providing services at the PHC level, there has been some upward task-shifting, with functions previously performed by lay health workers now falling within the responsibilities of paramedical professionals. For example, in Nepal immunization used to be the responsibility of village health workers, but is now primarily done by more professionalized auxiliary health workers.

Table 2 provides examples of common roles of CHWs in national programmes. Paper 5 in this series [22] explores the roles and tasks of CHWs in greater detail. In the first three roles shown in Table 2, the CHW potentially plays more than only a service delivery role; s(he) may also be an ***intermediary*** between the community and the health system. For these functions, effectiveness can be enhanced if the CHW is not only a service provider integrated within the healthcare system but also an embedded member of the community. This may be less important for the remaining functions. Note that the roles outlined here reflect current CHW programmes; as the roles of CHWs and indeed the healthcare system as a whole continue to evolve, we can expect further evolution in the content of CHW work.

**Table 2:** Common roles of CHWs in national programmes (with examples)

Role	Examples
Health educator, health promoter, behaviour change agent:	
Individual level	Family planning; maternal, infant, and child health, and nutrition
Community level	Community-led total sanitation and other water and sanitation initiatives; facilitating women’s groups
Adherence-support counsellor	For those taking medications for treatment of TB and/or HIV
Link community members to health services	As “navigator” or “*accompagnateur*”; including screening/case-finding and referral (e.g., for malnutrition) and default tracing (e.g., for HIV and TB patients)
“Depot-holder”, dispenser of health commodities	Oral contraceptive pills, oral rehydration salts, and zinc for child diarrhoea
Campaign field worker	Polio national or subnational immunization days or other supplemental immunization activities, child health days, mass distribution events for insecticide-treated nets or ivermectin (e.g., using a community-directed interventions approach)
Vaccinator	Routine child immunization and tetanus immunization as a component of antenatal care
Disease surveillance, case finding, reporting, and referral	Malaria, acute flaccid paralysis/polio, epidemic diseases (Ebola, Zika, COVID-19)
Limited clinical functions (otherwise performed by physicians or nurses), under task-shifting arrangements	Management of childhood illness, family planning, clinical procedures (e.g., injectable contraceptives, inserting contraceptive implants), diagnostics (using rapid diagnostic kits), childbirth care (antenatal care, formerly traditional birth attendant programmes, more recently community midwife or “skilled birth attendant” services)
Assisting health professionals, either during outreach activities or at fixed health facilities	Mobilization and site-level logistical support for routine immunization outreach
Enumerator, recording data	Registration of pregnancies, births, and deaths; assisting with civil registration; surveillance reporting for notifiable communicable diseases (including outbreaks)

Once CHW services become reasonably well established, there is a tendency to add new functions, responsibilities, and tasks. For example, Ethiopia’s HEWs began with responsibilities related to 16 different programmes (mainly prevention- and promotion-related). However, over time, numerous other responsibilities have been added, resulting in the “overloading” of a CHW, with the inevitable result that certain tasks end up deprioritized. Even if policy-makers and programme planners intend that CHWs focus primarily on health education or health promotion, communities tend to perceive greater value in clinical services, so, in turn, CHWs tend to prioritize what is most valued by their beneficiaries. There is generally higher status accorded to a role more closely approximating that of a physician, and in addition, members of the general population may perceive CHWs as offering second-class quality of care and opt to bypass them to seek care from providers they believe to be more qualified. In brief, although in most programmes it is not expected that CHWs play a role of social or political change agent, commonly they do have a role in health education/health promotion and act as intermediaries between health services and the community. Across programmes, there is a diverse range of specific functions, roles, and responsibilities that have been assigned to CHWs.

## 2) CHWs, lay or professional?

**Table:** 

Key message 4		
Any given CHW programme can be understood as falling at a particular point along three related but distinct dimensions: lay to professional, unpaid to salaried, and part-time to full-time		

### Lay to professional

In general, where a particular cadre falls along the lay-to-professional axis correlates with duration of pre-service training and licensing. CHWs who receive no more than a few days or weeks of training fall at the “lay” end of the spectrum, whereas CHWs with 1–2 years of training, or more, can be seen as falling towards the professional end. When these health workers are licensed by a formal accreditation body, they may be considered to be *mid-level health workers* rather than CHWs. Issues of training are explored further in Paper 6 in this series [23].

### Unpaid to salaried

The second axis concerns remuneration, which ranges from volunteers who do not receive any to service providers who are in formal employment and receive a salary and other financial benefits (e.g., a pension). We explore issues of remuneration in Paper 8 in this series [25]. Between these extremes, there is a wide variety and range of remuneration packages including monthly honoraria, travel or meal allowances (per diems), and performance-based incentives based on volume of certain services performed (e.g., accompanying a woman to a hospital to give birth). In some programmes, the total value of such allowances and incentives may approximate salaries at the low end of the civil service pay scale [36, 43]. These various arrangements reflect differences in CHW roles and responsibilities, in the settings and responsibilities, and the settings in which they work, as well as in factors such as the value placed on volunteer work, the extent to which being a paid government worker is viewed positively, and whether CHWs are able to control when they work.

One shared characteristic across all LMICs is that the majority of the working-age population is employed in the informal sector. In such an economic context, most CHWs may not have the expectation of salaried, formal-sector employment. They certainly, nonetheless, need to support themselves and their families. There are often opportunity costs associated with CHW work: time spent on CHW duties is time unavailable for other income-generating activities. There are also many examples of CHWs spending their own money to perform their role without being compensated, such as transportation expenses to help a patient obtain healthcare at a facility or needed medication [44].

### Part-time to full-time

The third axis refers to the number of hours or days a CHW works. Some CHWs have only occasional involvement, for example, participating in semiannual campaign-style outreach activities such as child health days, while other CHWs are full-time workers. Between the extremes on the continuum, there are cadres that do not work full-time but are engaged in CHW work almost every week and, in some programmes (and for some individual CHWs) on a close to full-time basis.

It must be noted, however, that where a particular cadre falls on any one of these axes is not perfectly correlated with where it falls on the other two.

### CHW definitions

Most definitions used over the past decade have defined a spectrum of CHW cadres from volunteers with brief informal training to paid, professionalized CHWs, with up to 2 years of formal pre-service training (as illustrated in Box 1, below).

Box 1. CHW definitions in the global literature

**Table:** 

The “lay health workers” in the Cochrane review by Lewin and colleagues (first published in 2005) [45] comprise CHWs at the less professionalized end of the spectrum, who “perform functions related to health care delivery, have been trained in some way in the context of the intervention, but have received no formal professional or paraprofessional certificate or tertiary education degree”. They could be paid or voluntary. Thus, this definition puts the CHW on the service delivery side (see Tension 1), but excludes those with more professionalized credentials In a review published by WHO in 2007 [46], it was stated that “CHWs …should: • be members of the communities where they work, • be selected by their communities, • be answerable to the community for their activities, • be supported by the health system but not necessarily a part of its organization, and have shorter training than professional workers”. The International Labour Organization (ILO) (2008) [47] described CHWs as: “provid[ing] health education and referrals for a wide range of services, and …support and assistance to communities, families and individuals with preventive health measures and gaining access to appropriate curative health and social services. They create a bridge between providers of health, social and community services and communities that may have difficulty in accessing these services”. This definition puts the CHW more definitively onto the PHC service delivery team, with the responsibility of providing information and support. The ILO also included in their definition that CHWs are “paramedical practitioners, occupations requir[ing] formal or informal training and supervision, recognized by the health and social services authorities”.
In the ILO’s new International Standard Classification of Occupations (ISCO-08) classification [47], there are several categories of workers that can be considered CHWs. Categorization according to the ISCO occupational groups and official job titles used in a jurisdiction do not always cohere. In some settings, the term “community health worker” or a similar term is used to refer to health workers who, according to the ILO ISCO classification, might more appropriately be referred to as *nursing and midwifery associate professionals* (ISCO 3221 and 3222) or *paramedical practitioners* (ISCO 2240). Conversely, health workers who have a role and profile consistent with ILO ISCO category 3253 (community health workers) may be classified and termed differently in a country (for example, community health officer, promoter, aide, educator, or volunteer). Categorization as community health workers (employment code 3253) is based on the health worker *role*, not on training or credentials, and listed tasks includes home visitation, giving information, supporting clients to access services, dispensing commodities, and monitoring and collecting data In 2013, the Global Health Workforce Alliance and its partners issued a joint statement [48] in which the term “community health worker” is used to refer to a wide range of both volunteer and remunerated health providers working within the community. In the important recent WHO guidance on policy and systems support for CHW programmes [3], the ILO 2012 language is cited, but revised slightly, providing somewhat more detail on the content of CHW work but—like the ILO document—WHO guidance focuses on health promotion tasks of CHWs, their integration into PHC teams, and linking the community with the health system
In a 2018 review, Scott and colleagues [49] described “community-based health workers” as “based in communities (i.e., conducting outreach beyond PHC facilities or based at peripheral health posts that are not staffed by doctors or nurses), … either paid or volunteer, … not professionals and … having at least some training, but < 2 years”. In 2017, Olaniran and colleagues [50] carried out a systematic review of definitions of CHWs and concluded that: (1) CHWs have an in-depth understanding of the community culture and language, (2) they are given standardized job-related training of a shorter duration than health professionals, and (3) a primary goal of their service is to ensure culturally appropriate health services to the community. The authors propose three categories of CHWs, based on educational prerequisites and duration of pre-service training: (1) lay health workers with little or no formal education who are given a few days to a few weeks of training, (2) those with some secondary education and subsequent informal training, and (3) those with some secondary education and training lasting from a few months to more than 1 year

It is evident from the country programme case studies in the newly published compendium [18] that in most LMICs, there have been progressive improvements in levels of education over the past several decades, with the result that there has been an evolution of CHW programmes towards higher educational entry requirements, longer pre-service training, and an increasingly professionalized role. In principle, licensing bodies set standards for professional practice and serve a quality assurance function. Professional associations serve as a voice for their members, advocating for their interests. Although increasing professionalization may also serve the public’s interest, this is not always the case. In Zimbabwe, in the late 1990s, the professionalization of nursing—by phasing out enrolled nursing training (2 years) in favour of registered nursing training (3 years)—contributed to staff shortages in rural PHC services, as graduates of the 3-year training programme were less interested in working in such positions, preferring hospital-based employment (or emigration).

In some recent documents, greater than 2 years of pre-service training has been the threshold for workers to be considered *mid-level* health workers rather than CHWs. However, this needs to be understood as an arbitrary cut-off and one that is likely to change over time, as education standards continue to rise and there is increased professionalization across all occupational types. For now, it may be helpful to think of CHWs as falling into several major categories, along a lay/volunteer-to-professional spectrum, where—at the most professionalized end of the spectrum (what we are labelling “auxiliary health workers”)—they grade into what could be considered mid-level health workers rather than CHWs. Note that not all categories of CHW programmes fall neatly into one of the types shown in Table 3.

**Table. 3:** Categorization of CHW types

Type of CHW	Recruitment	Duration of pre-service training	Place(s) of work	Employment status	Time commitment	Notes
Auxiliary health worker	Not necessarily local	> 1 year	Health post (± some outreach)	Salaried (often civil service, may be transferable to other locations)	Full-time	Examples: Auxiliary nurse-midwives (ANMs) in India and Nepal, lady health visitors in Pakistan, community health extension workers in Nigeria, public health midwives in Sri Lanka, and enrolled nurses in various PHC systems Typically 2 academic years of pre-service training although some are longer, e.g., Nigeria’s community health extension workers) [51]. Commonly hired through some unit of local government or through the state or national civil service structure. Because most health workers in this category are not required to be locally hired, they tend not to be “embedded” in the community to the same degree as other types of workers more commonly labelled CHWs
Health extension worker	Local, generally at least primary-level education required	~ 1.5–12 months, provided post recruitment	Health post, usually with significant outreach Some, with home visitation	Salaried or equivalent	Full-time	Examples: Bangladesh’s family welfare assistants and health assistants and Malawi’s HSAs. Ethiopia’s HEWs fall at the dividing line between this category and auxiliary health workers (as secondary school graduates, given 1 year of pre-service training following their recruitment) Most such programmes initially intended that the CHW spend most of her/his time outside the walls of a formal structure; however, in many programmes, CHWs have gravitated towards providing most services from a health post, subcentre, or dispensary. Salaries may be approximately equivalent to government primary school teachers or a little less
Community health volunteer	Local	A few days to a few weeks	Own home, beneficiary’s home	Non-salaried, may or may not receive financial (or other material) incentives	Occasional to regular part-time	Examples: Niger’s *relais*, Ethiopia’s Women’s Health Development Army, Nepal’s FCHVs Includes a spectrum, by level of time commitment, which we divide into 1) “regular”, usually with at least some activity every week but not full-time, and given up to several weeks of initial training, as well as continuing short episodes of in-service training; and 2) “occasional” or episodic volunteers, having relatively light, intermittent commitment, and given minimal training

Some programmes having what we are describing as “regular volunteers” make heavy use of allowances, per diems, and/or performance-based incentives. These volunteers are normally from, and live within, the communities they are serving. They are not considered to fall within formal sector employment. Although their role consists primarily of health education and linking people with health services, their functions may include dispensing (e.g., birth control pills, condoms, mosquito nets, and micronutrient supplements) and case management of childhood illness. In rare cases (e.g., Madagascar), they may give injectable contraceptives. In some programmes, duties and terms of service of regular volunteers start to approach those of HEWs (see Table 3), with significant part-time involvement (e.g., 10+ hours per week, as is common for accredited social health activists [ASHAs] in India) and financial incentives representing an important source of income. These may be performance- or commission-based; in some programmes, community health “volunteers” receive honoraria or allowances that could be considered de facto small salaries. In other programmes, although these CHVs perform regular functions, they normally put in less time (e.g., 5 h/week or less), and financial incentives may be minimal or not used at all.

In some countries, there are programmes with “occasional” volunteers who may be very numerous (e.g., the 3 million Women’s Development Army volunteers in Ethiopia). Typically, these episodic volunteers have functions limited to health promotion, though they may also be involved in periodic campaign-style events, distributing health commodities such as insecticide-treated nets, ivermectin (for prevention of river blindness/onchocerciasis), vitamin A, or vaccines (e.g., oral polio). Some countries in sub-Saharan Africa make use of such volunteers under a community-directed interventions model, in which the responsibility for leading and managing distribution—including selection and oversight of volunteers—rests with community leaders [52].

There are certainly categories of workers or volunteers that straddle the three categories we have identified here; as we have noted, Ethiopia’s HEWs fall at the boundary of our distinction between auxiliary health workers and HEWs, particularly with regard to duration of pre-service training. India’s ASHAs fall within the volunteer category, however in some parts of India, ASHA remuneration from allowances and performance-based incentives can approximate a salary [53].

In some local health systems, there are workers or volunteers corresponding to all the levels identified above; in many, one or more are missing. For any but the lowest of these cadre types, responsibilities may include support and supervision of lower-level cadres.

It may be most constructive to use terms and typologies descriptively (not prescriptively). Effective CHW programmes come in many shapes and sizes. The bottom line is: does it work and are arrangements fair and acceptable to those involved? But circumstances are continually changing. What was an effective model in the past will not necessarily be one in the future.

From our focus, under the first two tensions, on what roles CHWs play and how they can be defined, broader programmatic issues relevant for the organization and delivery of peripheral-level PHC services, making use of CHWs.

## 3) Government programme at scale or NGO-led demonstration project?

**Table:** 

Key message box 5	
In many public-sector CHW programmes, international NGOs play an important role; this can result in ambiguity with regard to perceptions of ownership or affiliationHow things work in relatively small, externally supported demonstration projects does not necessarily predict how they would work if implemented at scale, under routine conditions	

There are two related issues here: ownership/identification and scale.

### Primary identification with government or with an NGO? Who is running the show?

Of the programmes addressed in the case study compendium [18], several are well established and have been in existence for decades. The CHWs are paid civil servants, and financing for their support comes entirely from domestic government sources. Examples are the community health agents in Brazil [54] and the family welfare assistants/health assistants of Bangladesh [55]. Their continued viability and contribution is not dependent on external project-based donor support. However, there are other CHW programmes that are, in effect, only nominally government programmes, in which support from external partners is essential for their continued activity. Yet other programmes lie somewhere in between, where external partner support for in-service training, supervision, and other programme inputs may result in ambiguity concerning who exactly the CHWs belong to. Thus, there is a tension inherent in coordination, partnerships, and governance—as addressed in Papers 2 and 3 of this series [19, 20]—between the strategy for a CHW programme being driven by a national or subnational government or by development partners and donors.

Under these conditions, there are a variety of potential threats. How do inputs from external partners either contribute to or undermine the capacity of government PHC services and systems as well as the leadership role of government? We discuss this further in the papers in this series on planning, coordination and partnerships [19], governance [20], and financing [21]. How can sustainability be ensured? Who determines what should be prioritized? If the donor-supported partner is especially interested in seeing a particular intervention scaled up and delivered by CHWs, what impact could that have on other tasks the government expects of the CHW? What kind of engagement by international NGOs (INGOs), local NGOs, and faith-based organizations with government PHC services and programmes is most productive (and what practices should be avoided), with regard to both short-term gains in coverage on key interventions and longer-term strengthening of PHC services and systems?

Part of this tension has to do with the relationship between the government (at national, regional, district, and local levels) and its major development partners. To what extent does the government insist on, and development partners appropriately recognize, the government’s leadership role in setting priorities and making strategic choices, including with regard to PHC services? To what extent is the health system decentralized so that more local authorities can make context-specific decisions regarding their programmes and services and the role of development partners? These issues are also discussed in Paper 2 in this series on planning, coordination, and partnerships [19].

When resources directly available to governments are very limited, the added resources partners bring to the table can tilt priorities in directions favoured by the partners. For programmes supported by partners, often there is a felt need for some field-level worker, for example for HIV- or TB-related work, for nutrition, or for maternal/child health. Partners may then invest in supporting just these specific elements within the local health system, without regard for consequences on the broader range of services to be delivered. The best-funded programmes will then tend to bias efforts of the PHC system in the direction of their particular interventions. Moving from conditions of relatively heavy dependence on donor support to ownership by the government, there is a generally a need for (1) adequate, mainstreamed system supports, and (2) strong leadership/management. A viable, sustainable CHW programme will not be feasible—generally speaking—if implemented as part of a weak, inadequately resourced, peripheral-level PHC service. CHW programmes as add-ons or afterthoughts will not contribute much, at least not on a sustained basis. Work done by CHWs needs to be understood as part of the core work of the government’s PHC system.

Although there are circumstances in which INGOs have played and will continue to play a very constructive role contributing to ensure delivery of key services at the peripheral level of PHC, their support is often focused on comparatively narrow, mainly clinical areas (including preventive services such as immunization). Furthermore, by the nature of the funding, there is an incentive for INGOs to promote and support adoption and scale-up of new interventions, tools, and programmes. This often results in overloading, in the sense of multiplying the number of activities, interventions or vertical programmes that existing CHWs are expected to support. In practice, the problem is most often that other activities get crowded out, and the actual, on-the-ground prioritization by CHWs, as “street-level bureaucrats” [56], may not necessarily correspond to what is most likely to produce a significant population health impact.

On the other hand, there are many circumstances where NGOs play an important role at the district or subdistrict level by responding to specific needs of the peripheral health system, especially in conflict areas, areas with disease outbreaks, and those with hard-to-reach populations. They may also be instrumental for programmes in which CHWs play a social activist role by seeking to reduce inequities in health or address the social determinants of health. In some settings with fragile health systems, such as in Afghanistan, governments contract with NGOs at the provincial level to support delivery of the government’s PHC strategy.

### Small-scale, demonstration project or national-scale programme?

Certainly, findings from studies of comparatively small-scale, externally supported programmes can have relevance for nationally scaled programmes. However, efforts to introduce and scale up apparently successful small-scale models often result in disappointing performance. There are two related stumbling blocks: first, merely because something has worked well on a small scale, with dedicated resources, does not necessarily mean there is any realistic prospect of achieving similar effectiveness when one attempts to implement something similar at large scale, under routine programme conditions. Second, even if a model has been quite successful at scale in one setting, this is no guarantee that the same success can be replicated in a different context with different health, political and community system arrangements. The success of any given approach is rarely due only to the intervention; invariably, characteristics of the local system and context also matter.

One common scenario is an NGO-piloted CHW programme that the NGO, in turn, seeks to have the government adopt, scale up, and institutionalize. Sometimes this can be successful. Over the past 50 years, there have been a variety of highly influential, small-scale CHW programme experiences developed and led by NGOs or university-based groups. These experiences have served as the inspiration for important global initiatives in community health. For example, the 1978 Declaration of Alma-Ata was inspired in part by such experiences [57]. Similarly, today, recommendations are made to MOHs and donors, calling for large-scale, public-sector CHW programmes based on experiences with much smaller, more intensively supported programmes.

But there are also counterexamples. An example of the disconnect between successes observed in well-supported, small-scale programmes and efforts to implement similar programmes at large scale is the intensive postnatal, home-visit approach pioneered by Bang and colleagues in Maharashtra, India [58]. Based on this documented success and a few other relatively small-scale, intensively supported RCTs and demonstration projects, in 2009 UNICEF and WHO jointly issued a call to MOHs [59] to introduce such programmes at scale. Such programmes were subsequently widely adopted, but none have achieved high rates of effective coverage (and therefore population-level health impact) [60]. The translation from a successful small-scale demonstration project to an effective large-scale programme is not straightforward; it takes time and continued nurturing. And in some instances, the translation to scale will not be feasible. It is crucial to understand the conditions necessary for successful implementation of a particular approach and what it would take to meet and sustain these conditions at scale [61]. For successful large-scale CHW programmes, it is critical to develop adequately robust organizational support, including information systems, programme-commodity supply chains, management, supervision, and quality oversight. Lack of attention to these dimensions has resulted in lost opportunities for programme impact in many CHW programmes, as is evident in some of the compendium case studies [18].

## 4) Standardized or tailored to context?

**Table:** 

Key message box 6	
Tailoring a programme to context may help to it works well and meets local needs. But in large MOH programmes, pragmatic management considerations can push things in the direction of standardization	

Recently, there has been renewed interest in CHW programmes at a global level, with new or revised programme guidelines [3] and planning tools [62].^2^ But elaboration of such guidelines introduces an inevitable tension.

It is true that those responsible for developing the current major guidance documents have generally been careful to nuance their language to acknowledge the need for contextualizing application of their recommendations. Prudent decision-makers at the country level will take such recommendations into consideration but, at the end of the day, exercise their own judgment—based on an understanding of their own programmes and key contextual factors—to determine programme approaches appropriate to their setting. Papers 2 and 3 in this series [19, 20] address these issues further. There is a great diversity of contextual factors potentially relevant for CHW programmes. In some settings, NGOs and the private sector are engaged in direct PHC service delivery. Civil society entities, such as Red Cross/Red Crescent, may play an important role. Circumstances can differ enormously between informal urban settlements, rural areas, geographically remote populations, and pastoralist groups—each calling for different approaches to PHC, and different roles and strategies in the use of CHWs.

Countries differ markedly with regard to the degree to which management of PHC services is centralized or decentralized. Some PHC services have a hierarchical management structure reporting up to a national MOH. In other countries, the responsibility for PHC financing and delivery rests primarily with local government, with a minimal role for national or subnational levels of government. Such differences in governance have important implications for how standardized PHC services are likely to be (see Paper 3 in this series [20]).

So how standardized or tailored should CHW programmes be? And how does that vary by setting? Table 4 provides a framework for considering these questions.

**Table. 4:** Consequences of standardized and tailored approaches to CHW programming

Standardized	Tailored
Simple, neat, comprehensible Easy to monitor and report on Fits a top-down, technocratic mindset Easy for planners There may be more consistency on important technical aspects Less likely to be appropriately responsive to the situation on the ground	Empowers local managers/decision-makers Likely to be a better fit with actual needs More scope to *adapt* to context based on observed performance and changing circumstances Fits with devolved, decentralized systems for governing CHW programmes [20] Uncomfortable for global- and national-level planners, policy-makers, donors

A hierarchical, technocratic, managerialist perspective has the virtue of apparent simplicity and visibility [63, 64]. This makes it inherently appealing for global-level agencies, technical assistance groups, donors, and centrally based officials and technical officers in MOHs. There have been calls for standardizing definitions and roles for CHW programmes [2]. Indeed, it complicates the lives of those trying to write about CHW programmes or trying to count CHWs across multiple jurisdictions when there is such diversity on the ground. Furthermore, for both governments and donor-supported partners, efforts to scale up programmes are much simplified by using a single set of specifications.

Because different CHW programmes have different goals and operate in a wide range of settings, specific choices that work well or are essential in one particular setting are not necessarily helpful in another. Flexibility and adaptability can often result in more responsive PHC services, including those delivered by CHWs, and can enable changes and improvements in programme design when it becomes evident that initial designs are falling short of the goals established. Workers and managers need sufficient decision space to make choices that adequately respond to the situations they are actually dealing with. Furthermore, health programmes are implemented in complex, dynamic circumstances; for programmes to be effective, they need to be well tailored to those circumstances, and adaptive as circumstances change.

## 5) Horizontal or vertical?

**Table:** 

Key message box 7	
In many settings, CHWs focus on delivery of a small number of interventions, associated with currently prioritized health programmes (notably, maternal and child health, family planning, HIV/AIDS). But broad improvements in health and well-being will require more than delivery of a small number of prioritized interventions. Programme content at this level should be driven by what will produce the greatest population benefit	

As discussed earlier in this paper, even before the first identifiable CHW programme a century ago, there were competing visions on how to do public health; one segment of the global public health community advocated for more broadly based approaches to health and well-being—including an emphasis on socioeconomic determinants of health and the need for robust primary-level preventive/promotive/curative services available to all—and another segment focused on delivery of effective, high-impact interventions at high coverage, aiming for population-level control of specific (mainly communicable) diseases. This tension was evident in the controversy through the 1980s and 1990s that pitted those calling for “comprehensive PHC” against advocates of “selective PHC” [65, 66]. Since then, some leaders in global health have sought to build a case that this tension can be resolved, addressing broader health determinants, building local systems, *and* ensuring high-coverage delivery of efficacious interventions [67]. Papers 2 and 3 in this series [19, 20] address these issues further.

The same tensions seen between more comprehensive, integrated PHC services and those focused on delivery of single interventions [65, 68] are also seen in CHW programmes. There are programmes in which a single CHW is responsible for the full range of services provided at the most peripheral tier of the PHC system (e.g., Ethiopia’s HEWs, providing curative care as part of their work in health posts). In other settings, specific CHWs may have a narrower, more specialized role. In yet other programme contexts, there may be more than one category of CHWs working in the same community, with some division of labour. For example, one cadre (primarily male) may be responsible for immunization outreach and another (primarily female) for family planning and antenatal care. Examples include: the family welfare assistants and health assistants in Bangladesh [55], the male and female pairs of *binômes* of Rwanda [69], and the now phased-out village health workers and maternal child health workers in Nepal. Such gender-based role differentiation is important, in some cultural contexts [32], in determining the effectiveness of CHWs whose responsibilities include maternal/child health and family planning, for example.

In many countries, CHWs are involved in periodic, intensive, outreach campaigns offered annually or semiannually. Such models include supplementary immunization activities, [70, 71], national or subnational [polio] immunization days [72, 73], child health days [74, 75], and community-directed interventions [76]. These periodic outreach activities typically deliver one or a very small number of interventions (e.g., polio vaccine, vitamin A, or long-lasting insecticide-treated nets) and seek to achieve high population coverage. Some critics have pointed to the tradeoffs this may entail, distracting those in the peripheral-level PHC system from routine services they would otherwise be delivering [77]. Can campaigns and routine delivery be complementary strategies, or do campaign-style efforts necessarily undermine and weaken routine services?

One consequence of vertical efforts superimposed on more integrated peripheral-level PHC services has been the creation of different kinds of CHWs working in the same setting. A number of countries have CHW cadres originally created for family planning services or as field vaccinators. More recently, special categories of CHWs have been created as adherence counsellors for HIV and TB programmes. Sometimes these categories of CHWs come under the same management structure, but in other settings they may report through separate management structures all the way up to the central MOH. Particularly where CHWs are managed under separate programmes, funding levels and logistical support may differ between programmes, having negative effects on the less well-resourced programmes and their CHWs, with consequences for CHW motivation and retention.

In global health, there has been a major focus on achievement of high population coverage for specific, mainly clinical, preventive and curative interventions (promotion of exclusive breastfeeding is an exception, being considered a behavioural intervention), associated with categorical funding and vertical delivery systems. Associated with this there have been genuine successes, notably immunization, which can legitimately claim credit for substantially reducing child mortality. However, arguments have been made [34] that an approach focused on specific interventions has tended to result in relative neglect of routine services (i.e., the broader set of services and functions of PHC) and, at a minimum, has represented a missed opportunity for strengthening systems and delivery of services in PHC. Frenk makes the case that it does not have to be either/or; it is possible to simultaneously build strong systems and service delivery, and ensure high coverage for the most impactful interventions [68].

## Discussion

CHWs perform a wide variety of roles in peripheral-level PHC, varying by country setting, as discussed elsewhere in this series [22]. Many CHW responsibilities overlap with those of other health workers, some falling into the category of *task-shifting* (i.e., functions performed, in other contexts, by physicians, nurses, or other more credentialed health workers). In most LMICs, the general standard of education has been increasing over the past several decades. Consequently, across all occupations, there has been some movement towards higher education standards at entry and longer periods of pre-service training, as discussed elsewhere in this series [23]. This professionalizing tendency is evident in the health sector and is reflected in changes in CHW programmes. To some extent, this has already resulted in what could be seen as task *up*-shifting. For example, in settings where lay health workers with only elementary education were, until recently, responsible for immunization, this function is increasingly being assumed by health workers with formal post-secondary nursing or paraprofessional training. It can be expected that in the future, this trend of up-shifting tasks to more highly trained staff will continue as the number of more highly credentialed health workers increase. With such changes, how can the benefits of having someone in an intermediary role between health services and the community be maintained? In what ways may we see a progressive professionalizing of the intermediary role of care navigator or case manager? What continuing role could there or should there be for volunteers and lay workers?

In this paper, we have noted two, closely related tensions in global health work, more generally, that also apply to CHW work, notably (1) government versus donor/INGO ownership or influence on CHW programme work, and (2) the relationship between—on the one hand—relatively well-resourced pilot or demonstration activities implemented by an NGO and—on the other—delivery of services at scale, under routine programme conditions. The dynamics among governments, donors, and other development partners play out differently, depending on the setting and the specific actors involved. What constitutes good development practice has been well articulated in the past, for example in the Paris Declaration on Aid Effectiveness [78] and the Accra Agenda for Action [79]. Nevertheless, until the problems that these international statements address are fully resolved, we will continue to see dysfunction in the relationships between key actors that undermines effectiveness and sustainability of PHC services and programmes, including those in which CHWs play an important role. These issues are further examined in the paper on financing of CHW programmes in this series [21].

One of the challenges with pilot and demonstration projects is that, in many instances, they are developed and implemented by external partners with resources not likely to be available on a continuing basis, thereby undermining the likelihood of these efforts being sustained. Such cases are complicated by the dynamic we have noted between governments, donors, and other development partners. But, even when a pilot or demonstration project is entirely “home-grown”, there is frequently a failure to appreciate that the results achievable at small scale with charismatic leadership, dedicated staff, and adequate resources do not reliably predict results that can be achieved implementing at scale under routine programme conditions in the public sector. Nevertheless, if a donor or national government commits to and actively supports a particular intervention or programme model—in the hope of replicating results from a pilot—there can be considerable momentum driving scale-up forward, regardless of whether or not the initiative is delivering its intended benefit. The bigger a programme becomes, the greater the reputational costs of acknowledging performance problems. Paper 10 in this series [27] reviews CHW programme performance and its measurement. The history of CHW programmes over the past several decades is littered with examples of programmes that have gone to scale and been sustained over years, despite evidence of poor performance [80]. All actors in global health need to be on guard against uncritically endorsing continuing, large-scale programmes in the absence of evidence of impact [80].

As reflected in the Disease Control Priorities initiative, now in its third major iteration [81], there has long been a strong technocratic element in global health development work, emphasizing delivery of specific interventions for which there is robust evidence of effectiveness in reducing the burden of the principal causes of mortality and morbidity in LMICs. Major donors, including World Bank, the US Agency for International Development, the Bill & Melinda Gates Foundation, and others continue—to a large extent—to align their efforts with such a perspective. Unquestionably, this approach has a history of major successes, including the eradication of smallpox, more recent major reductions in the burden of immunization-preventable diseases, and the large-scale delivery of antiretroviral medications for the treatment of HIV infections in sub-Saharan Africa. But, arguably, there has also been a cost to the prominent place this global health philosophy has occupied. As Packard has argued [38], this has to varying degrees crowded out efforts to strengthen basic PHC services, to address the socio-behavioural drivers of health outcomes, and involve communities in the strengthening of their own health systems.

Frenk [68] makes the case that the two areas of concern can be reconciled, putting serious efforts into building a strong peripheral-level PHC platform, which can in turn serve as the vehicle for delivery of a broad range of health interventions that address priority health problems, including those that may not be prioritized by donors. For example, with a more robust peripheral-level PHC system, CHWs could play an important role such as identifying and initiating treatment of hypertension and other important and increasingly common noncommunicable diseases. Perry [82] has laid out a vision for PHC in the twenty-first century that incorporates selective, vertical interventions along with community engagement on a common platform of peripheral health services, in which CHWs are key actors.

That brings us to the question: what place is there for CHWs in the future of PHC? First, it must be acknowledged that much will change. Almost everywhere, we are seeing rising levels of education and living standards and—with them—rising demands for better and more accessible healthcare services (as reflected in the call for UHC). Almost all societies are seeing increasing urbanization, which will certainly have consequences for the future of PHC, including CHW programmes.

Although PHC efforts in LMICs will certainly need to be directing more attention to noncommunicable diseases, the current COVID-19 pandemic is a reminder that we have not altogether left communicable diseases behind. Indeed, as health-sector leaders in Liberia and Sierra Leone learned as they responded to Ebola, reliable early detection and timely response to outbreaks is a key function that needs to be functional at the most peripheral level. Recent experience with COVID-19 has carried this lesson to a much wider audience, bringing with it a recognition of the critical role CHWs can play in disease surveillance/outbreak detection, and in a variety of other important, community-level roles [10]. The future will bring important epidemiologic and demographic transitions. Increasingly, PHC services in LMICs will need to address noncommunicable diseases and the health problems of older adults.

Historically, government PHC services have primarily targeted rural populations. In most LMICs, there has been a trend over the past several decades of rapidly increasing urbanization, which is likely to continue. Municipal governments have generally been responsible for a range of public health functions including water, sanitation, solid-waste disposal, and other environmental health issues. However, to date, government PHC services have been limited in urban areas and have largely focused on a comparatively small number of clinical preventive services (e.g., immunization, TB control). Unlike rural areas, where government PHC service may play an important role in curative services, in urban settings such a role commonly falls more to private-sector providers. In this more complex, pluralistic service delivery environment, what role is there for government PHC services in general, and for CHWs more specifically? Brazil offers a model of multidisciplinary, generally physician-led, urban community health centres, in which CHWs have an important role to play as intermediaries between health services and the community [54].

## Conclusion

In this paper, we have discussed the variety and complexity of CHW programmes and the PHC systems of which they are a part. It is likely that the tensions we have described will continue to evolve in the coming decades, shaped by changing implementation contexts and the increasingly wide implementation of digital health interventions [83]. However, an enduring message is that those planning and managing these programmes need to avoid overly formulaic, standardized approaches that fail to adequately take local into account [83]. Local-level health systems are complex, human systems comprised of a range of actors, in interaction with one another, each with perspectives, needs, values, and interests. Efforts to strengthen local health services and systems need to engage respectfully with these various actors and—at the same time—to attend seriously to population-level effectiveness (including ongoing performance tracking and adaptation in response to observed performance and evolving circumstances).

For improvements in population health outcomes, we need robust, resilient, adequately funded, high-performing PHC systems. CHW programmes are now, in many countries, an increasingly important element in such systems, and it is likely that their importance—and our knowledge on how best to design, implement, and sustain them—will continue to grow. Paper 11 [84] summarizes the findings from this series and points the way toward the many changes that will need to be taken to achieve robust, resilient, adequately funded, high-performing CHW programmes.

## Data Availability

Any articles and other materials cited by the authors are available from the corresponding author.

## Abbreviations

ASHA: Accredited social health activist
CHV: Community health volunteer
CHW: Community health worker
COVID: Coronavirus disease
FCHV: Female community health volunteer
HEW: Health extension worker
HIV: Human immunodeficiency syndrome
ILO: International Labour Organization
INGO: International nongovernmental organization
LMICs: Low- and middle-income countries
MOH: Ministry of Health
NGO: Nongovernmental organization
PHC: Primary healthcare
TB: Tuberculosis

## Footnotes

1 Most LMICs are seeing significant urbanization; it can be expected that in the future, we will see an increasing number of large-scale public-sector CHWs functioning in urban settings.

2 Note that this discussion concerns *programme approaches* and *strategies*. The authors acknowledge that use of standardized *technical guidelines and tools* such as clinical protocols is less affected by context.
